# HF etched glass substrates for improved thin-film solar cells

**DOI:** 10.1016/j.heliyon.2018.e00835

**Published:** 2018-10-19

**Authors:** Hyeongsik Park, Doyoung Kim, Junhee Jung, Duy Phong Pham, Anh Huy Tuan Le, Jaehyun Cho, Shahzada Qamar Hussain, Junsin Yi

**Affiliations:** a College of Information and Communication Engineering, Sungkyunkwan University, Suwon 440-746, Republic of Korea; b Electronic Convergence Materials and Device Research Center, Korea Electronics Technology Institute, Seongnam 13509, Republic of Korea; c School of Electricity and Electronics, Ulsan College, Ulsan 680-749, Republic of Korea; d Department of Energy Science, Sungkyunkwan University, Suwon 440-746, Republic of Korea; e Division of Computational Physics, Institute for Computational Science, Ton Duc Thang University, Ho Chi Minh City, Viet Nam; f Faculty of Electrical and Electronics Engineering, Ton Duc Thang University, Ho Chi Minh City, Viet Nam

**Keywords:** Materials science

## Abstract

A hemisphere-array textured glass substrate was fabricated for the development of an improved thin-film (TF) silicon solar cell. The HF-H_2_SO_4_-etchant system influenced the light path owing to the formation of the strong fluorine-containing HSO_3_F acid. In particular, the etching system of the various HF concentration with a constant H_2_SO_4_ solution is related to make an improvement of optical transmittance and light trapping structure without a uniform pattern. According to the specular transmittance measurements, the haze ratio was maintained for the glass sample etched with 35% HF in the longer-wavelength region. The proposed substrate was implemented in a TF-Si solar cell, and an improved conversion efficiency was observed according to the short-circuit current density owing to the increase in the haze ratio. This morphology, therefore, induces more scattering at the front side of the cell and leads to an improvement of the open circuit voltage gain for the HF 25% cell. It will be helpful to understand the application of thin film solar cell based on the HF-H_2_SO_4_ etching system for the readers.

## 1 Introduction

Certain methods can be employed to improve open-circuit voltage (V_oc_), fill factor (FF), and short-circuit current density (J_sc_) for increasing the efficiencies of solar cells [1]. In particular, etching methods for both transparent conductive oxides (TCOs) and substrate based on light-trapping can be used to increase the optical path length, which in turn increases the current density of thin-film (TF) silicon solar cells [2].

The methods for changing the surface morphology of TCOs include wet, dry, and vapor etching, and a direct growing process can be used to form a light-trapping structure. The degree of reconstruction on the surface is determined by the haze value obtained from the ratio of the transmittance to diffuse transmittance as a function of the wavelength of light [3, 4, 5, 6, 7, 8, 9]. The improvement in the efficiency of a TF-Si solar cell such as a microcrystalline single junction or tandem cell has been proven to be extremely difficult when the TCO-etching method is applied, because of the weakness of scattering in the longer-wavelength region [10, 11].

To prevent the reduction of light loss in the longer-wavelength region of a TF-Si solar cell, an alternative is the adoption of the wet and vapor etching methods [5]. Even though random wet etching is a simple process, it is not recommended for achieving a uniform surface [11]. The UV-photolithography method, however, is advantageous for achieving surface uniformity because it is an attractive solution for the texturing of TF-Si solar cells owing to its low cost, minimized damage, appropriate imaging resolution, and high throughput. Although random wet etching with a hydrogen-fluoride (HF)-chemical solution is a simple and inexpensive method, a non-uniformly-etched surface is produced due to the remains of the insoluble product present in the chemical solution [12]. The HF-solution-based etching also leads to an increased haze value, which has an adverse effect on the improvement of the transmittance.

To improve both the haze value and transmittance, an analogous periodic-texture-etched surface has been proposed [13]. However, it is difficult to achieve the advanced optical performance of TF-Si solar cells without a photolithographic patterning process via wet chemical etching [14]. Some groups have reported the formation of a rough texturized surface through the utilization of an aluminum-induced texture based on wet chemical etching [15, 16]; however, a precise control of the first aluminum thickness and annealing conditions is required. To solve the aforementioned problem, this work proposes the formation of a geometrical structure composed of a hemispherical array, which is etched with a chemical solution. The HF/H_2_SO_4_ mixed-etchant texturing is based on a thermally exothermic reaction, and an analogous periodic-texture-etched surface is formed after the etching.

In this study, an analogous periodic-texture-etched substrate surface is fabricated via the random etching method, followed by the characterization of the surface morphology. The ratio of HF to H_2_SO_4_ was fixed at 1:5, and the added HF concentration was varied from 1% to 45%. Subsequently, a 560 nm-thick Al-doped zinc-oxide (Al_2_O_3_ 1 wt.%-doped ZnO) film was deposited onto the texture-etched surface of the substrate for the formation of the cell. The reduction of V_oc_ and FF was expected upon the completion of growth of the Si layer. In addition, in order to evaluate the power generation characteristics, surface reconstruction was applied to the substrate to fabricate the TF-Si solar cell.

## 2 Experimental

The preparation process for the light trapping on the glass substrate is as follows. The first (SC-1) and second steps (SC-2) are performed to remove the organic and inorganic elements on the glass surface. Prior to the etching, the substrate is dried in an oven at 100 °C–110 °C for 10 min to remove the water on the surface.

**Fig. 1:**
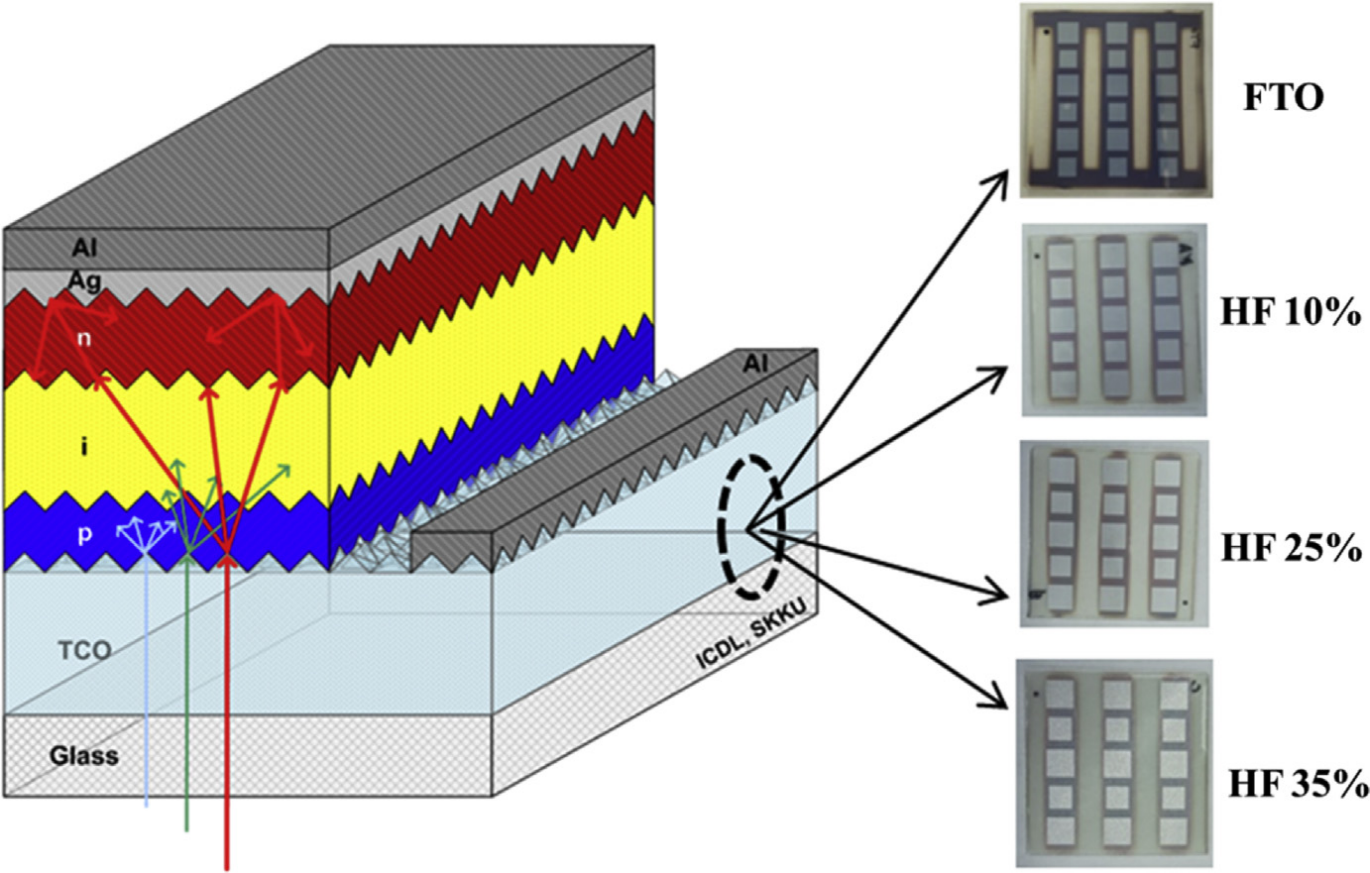
A schematic of thin film silicon solar cell with different 4-types of glass substrates.

A solution of HF (concentration of 49%, DUKSAN Chemical reagents) and H_2_SO_4_ (95% extra pure, DUKSAN reagents) is prepared at the fixed ratio of 1:5, and the HF concentration is controlled from 1% to 45% using deionized water. The total amount of the etching solution is 420 mL. For preparing the solution, the following information should be considered. First, a diluted HF solution should be poured into a Teflon container and thereafter poured into the sulfuric acid; the solution can be dangerous if it is mixed in reverse. As the mixed solution is reasonably hot in this instance, it should be cooled to room temperature. The glass substrate must be fixed onto the cleaning-cassette kit using Teflon tape to avoid disturbing of the sample during the etching. When the substrate is dipped into the solution, it starts to boil due to an exothermic reaction. After the etching process, the substrate is cleaned using the aforementioned SC-1 and SC-2 steps. AZO (Al_2_O_3_ 1 wt.%-doped ZnO) films are deposited on the etched-glass substrate using a radio frequency (RF) magnetron sputtering system. The working pressure of the sputter chamber, argon-gas flow rate, substrate temperature (T_s_), and angular speed of the substrate are fixed at 12 mTorr, 20 sccm, 300 °C, and 5 rpm, respectively. Initially, the sputtering chamber is evacuated with a base pressure of ∼10^−6^ Torr. For more effective step coverage, RF power of 300 W is used, and the total thickness is approximately 560 nm. The a-Si:H-TF solar cell is fabricated on the etched-glass/AZO film structure using a multi-chamber cluster plasma-enhanced chemical vapor deposition system as shown in Fig. 1 [17].

The surface morphology and root-mean-square (RMS) roughness of the etched-glass substrate were measured using a scanning electron microscope (SEM, Model: JSM6700F, JEOL) and surface profilometer (Model: D-500, KLA Tencor), respectively. The optical characteristics (total and diffused transmittance rates) were measured using a solar cell spectral response measurement system (Model: QE/IPCE QEX7, PV measurement, Inc.). The haze ratio of the etched-glass substrate was calculated using the ratio of the diffused transmittance to the total transmittance. The current-density and voltage of the a-Si:H-TF solar cell were measured under an air mass of 1.5 (100 mW/cm^2^) at room temperature. The spectral response of the a-Si:H-TF solar cell was measured using an external quantum efficiency (EQE) system.

## 3 Results and discussion

**Fig. 2:**
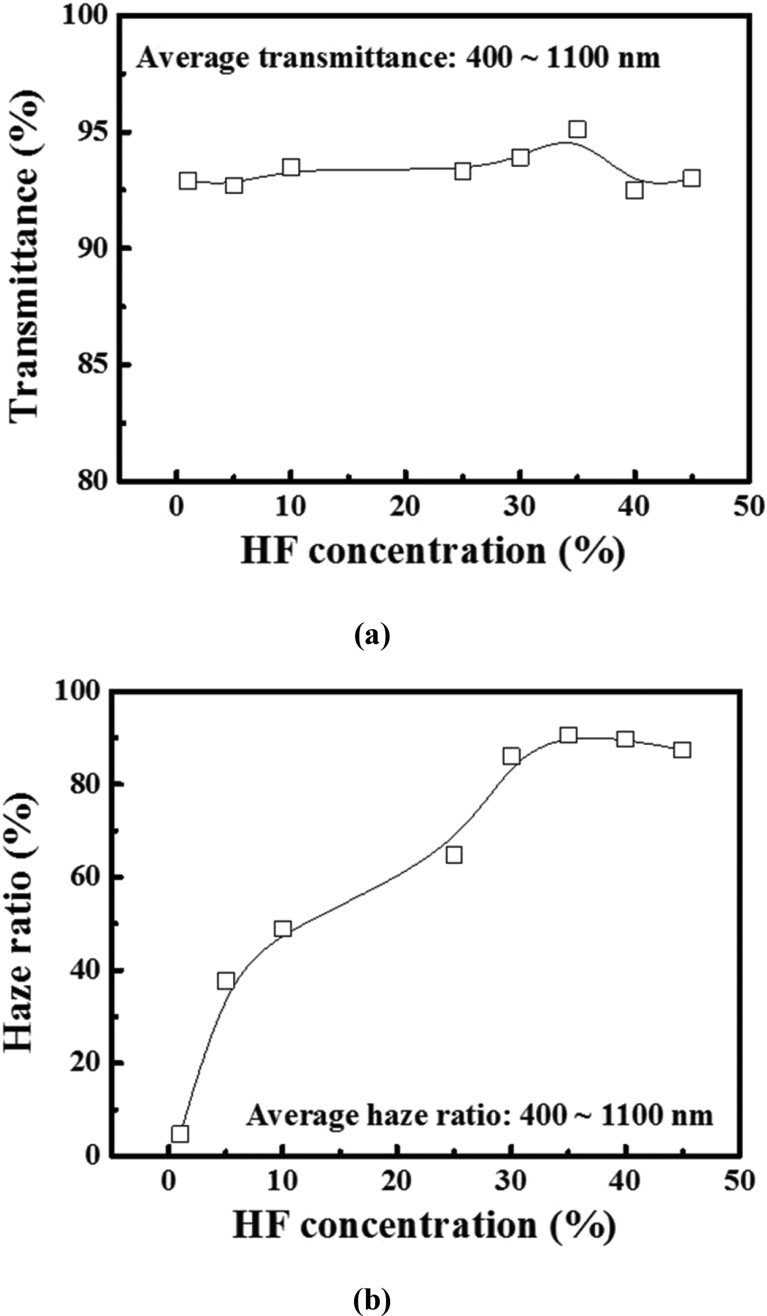
(a) Average transmittance and (b) haze ratio as a function of HF concentration. The HF + H_2_SO_4_ solution ratio was fixed at 1:5.

The optical properties at various HF concentrations are shown in Fig. 2. In the case of the glass texture-etched with the HF concentration of 35%, the transmittance and haze ratio are higher at 95.1% and 90.6%, respectively; this indicates that high HF concentration induces high transmittance and haze ratio after the etching process. The transmittance is constant for all the samples, whereas the haze ratio of the texture-etched glass is increased for higher HF concentrations as compared with the concentrations in the range HF 1–10%. This result indicates that the HF concentration plays an important role in the optical performance owing to the difference between the texturing mechanisms, thus facilitating the following discussion.

An HF consists of the corrosive hydrogen ion (H^+^), and the fluoride (F^+^) ion, thereby acting in the etching reaction. Here, the HF acid corrodes the glass surface and thus fluoride ion penetrates the glass. Once in the chemical reaction, fluoride ion binds themselves among other materials such as Ca, Na, K, Ba, Zn and thus generates the byproducts such as a H_2_SiF_6_, NaF, KF, BaF_3_, ZnF_2_ and CaF_2_ (Eqs. (2), (3), (4), (5), (6) and (7)) in the first reaction and thereafter some byproduct of SiF4 by a reaction with a SiO_2_ in the glass (Eq. (1)) is soluble or not soluble in the water.

(1) SiO_2_ + 4HF → SiF_4_ + 2H_2_O

(2) SiO_2_ + 6HF → H_2_SiF_6_ + 2H_2_O

(3) Na_2_O + 2HF → 2NaF + H_2_O

(4) K_2_O + 2HF → 2KF + H_2_O

(5) Ba_2_O_3_ + 6HF → 2BaF_3_ + 3H_2_O

(6) ZnO + 2HF → ZnF_2_ + H_2_O

(7) CaO + 2HF → CaF_2_ + H_2_O

The HF-H_2_SO_4_ etching system produces a higher roughness value, which can be ascribed to the formation of the strong fluorine-containing HSO_3_F acid [10, 18]. The HF concentration is continually maintained along with the changes in the sulfuric acid concentration, and this continues with the increase in the activation energy. The regeneration of HF occurs after the second reaction with H_2_SO_4_ (Eqs. (8), (9), (10), (11), (12) and (13)) during the etching process. The addition of H_2_SO_4_ is, therefore, effective for increasing the etch rate and surface roughness. These surfaces are less smooth than the mechanically polished ones, indicating that it is possible to transform ground surfaces into optically transparent surfaces.

(8) H_2_SiF_6_ + SiF_4_ + H_2_SO_4_ → 2SiF4 + 2HF + H_2_SO_4_

(9) 2KF + H_2_SO_4_ → 2K_2_SO_4_ + 2HF

(10) 2NaF + H_2_SO_4_ → Na_2_SO_4_ + 2HF

(11) CaF_2_ + H_2_SO_4_ → Ca_2_SO_4_ + 2HF

(12) 2BaF_3_ + 3H_2_SO_4_ → Ba_2_(SO_4_)_3_ + 6HF

(13) 2ZnF + 2H_2_SO_4_ → 2ZnSO_4_ + 4HF

**Fig. 3:**
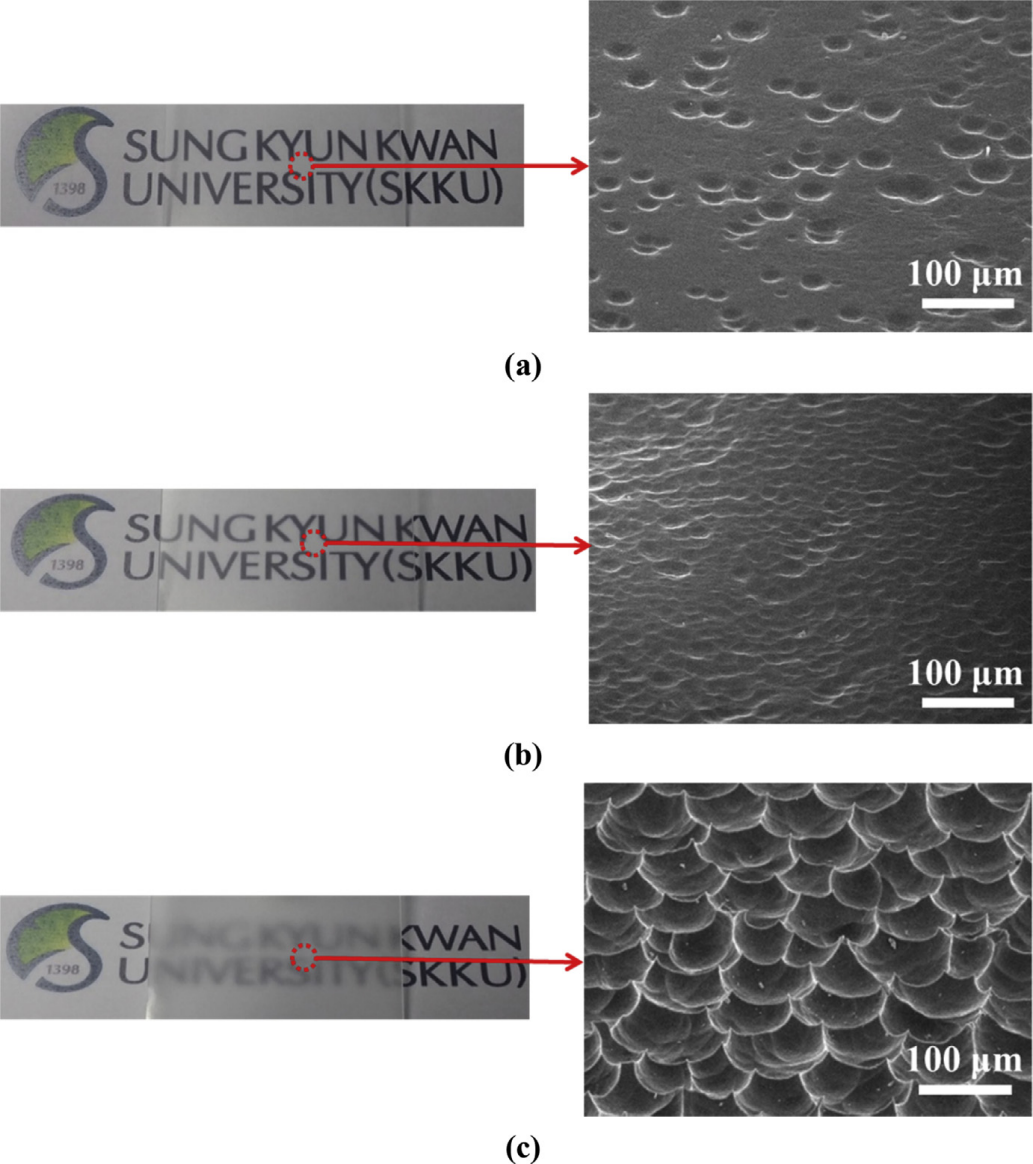
Photographs and tilted-view SEM images of the texture-etched glass with different haze value using an etching solution of (a) HF 10%, (b) HF 25%, and (c) HF 35% for thin-film silicon solar cell application. The angle of the tilted view is 30°.

Fig. 3 shows the photographs and SEM images of the texture-etched glass substrate for the fabrication of TF solar cells according to different haze value. For a HF concentration of 10%, the texture-etched glass surface features a sparse crater distribution, whereas the texture-etched glass surface for the HF etching concentration of more than 25% shows several hundred micro-sized craters. With the increase in the HF concentration above 30%, a few of the craters show an analogous periodic texture and the surface is uniformly etched as shown in Fig. 3(c). From this figure, it is evident that the HF 35% etched glass substrate has a higher haze ratio than both bare glass and the HF 10% etched glass substrate.

**Fig. 4:**
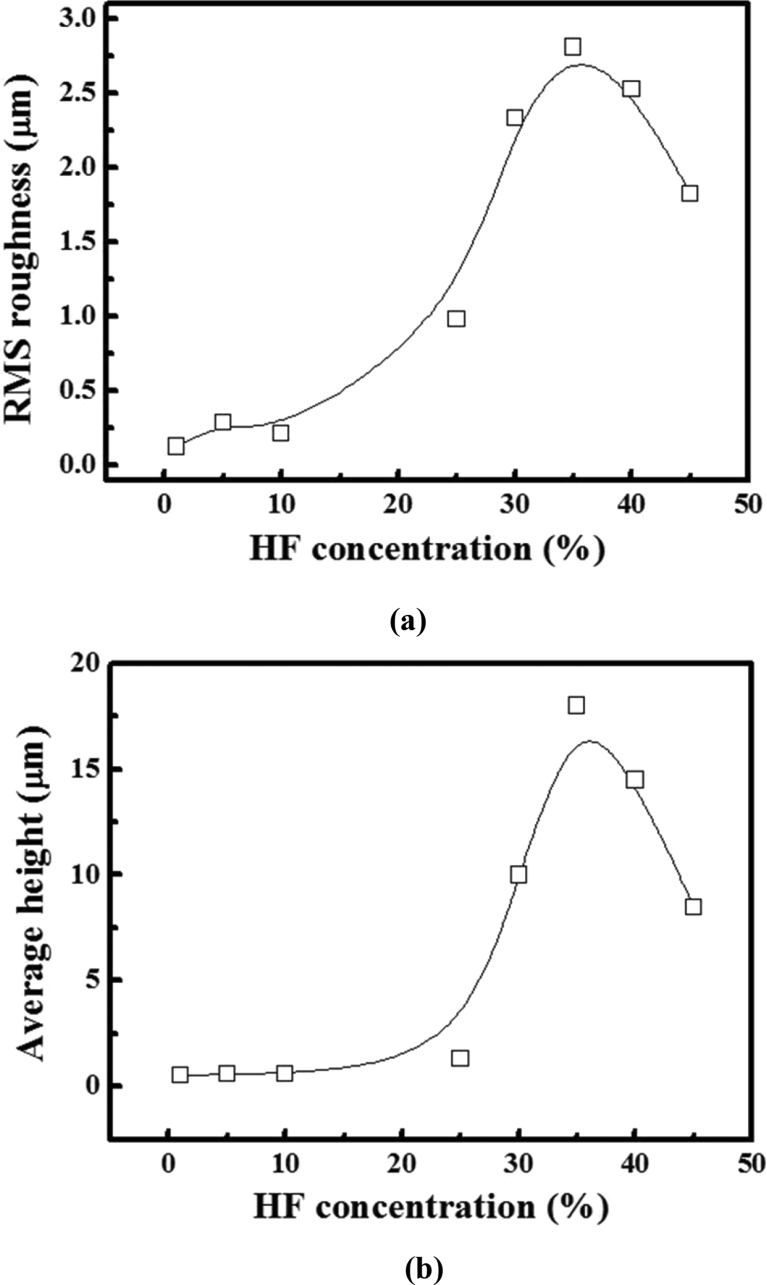
(a) RMS roughness and (b) average height as a function of HF concentration.

The difference in the surface morphology of the texture-etched glass can be observed in Fig. 4(a), where the RMS is shown after the scanning of the surface. Fig. 4(b) shows the changes in the average height with different HF concentrations. All the texture-etched glass substrates present a very high roughness up to 3 μm (HF concentration of 35%) with the increase in the etching time. The surface roughness for the HF concentrations of 10% and 25% is reasonably lower than that for the HF concentrations between 30% and 45%; this is explained by the different texturing mechanisms induced by the different HF concentrations, which is due to the higher quantity of OH^−^ ions in the etchant [11].

**Fig. 5:**
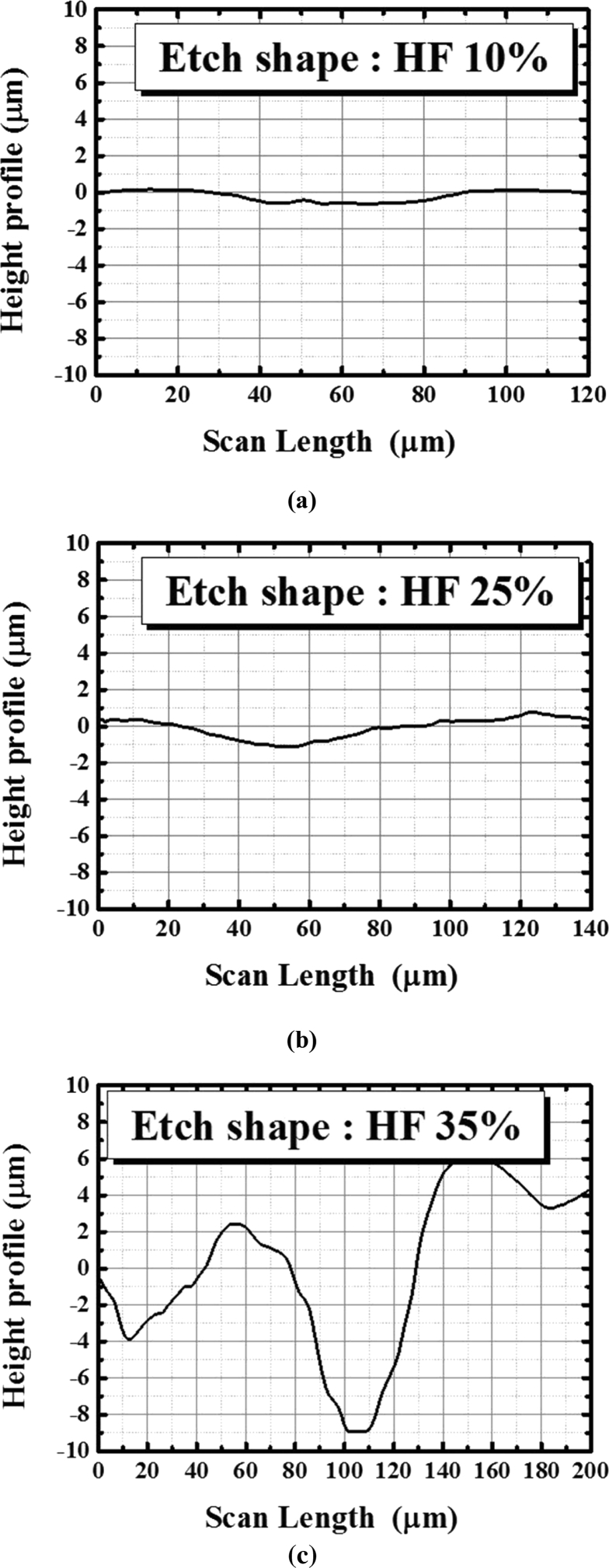
Height profiles of the three samples (a) HF 10%, (b) HF 25%, and (c) HF 35% of a texture-etched glass substrate for the fabrication of a thin-film Si solar cell.

The height profiles of the three samples (HF 10%, HF 25%, and HF 35%) are plotted in Fig. 5. It can be observed that the height profiles of the HF 10% and HF 25% samples are similar, which is expected from the corresponding texture-etched sizes. The height profile of the HF 35% sample is sufficiently rougher than those of the other samples. Here, the selection standards for the authors' sample are based on the texture-etched sizes of ∼0.6 μm, ∼1.5 μm, and 16 μm, respectively.

**Fig. 6:**
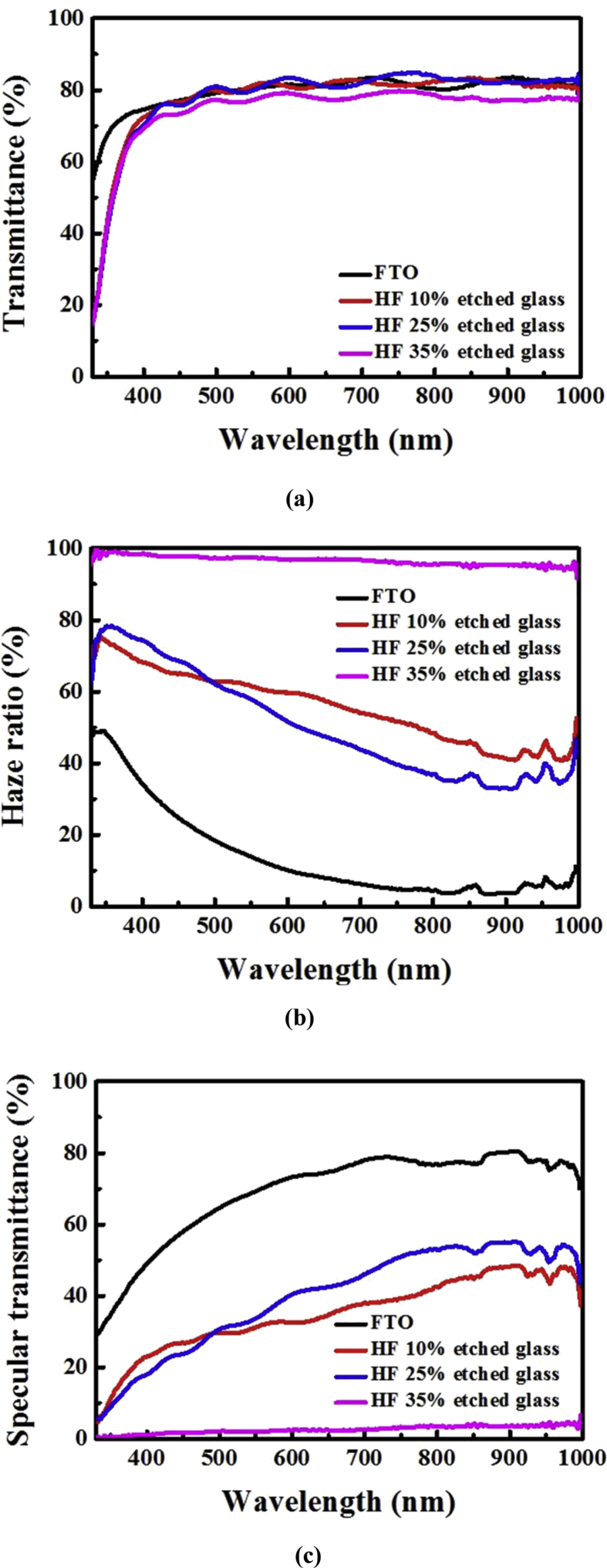
Optical properties of a double-layer ZnO:Al thin film: (a) total transmittance, (b) haze ratio, and (c) specular transmittance. The total thickness is approximately 560 nm.

Before the fabrication of the TF solar cell, the AZO film is deposited on the texture-etched glass substrate. The AZO thickness is fixed at approximately 560 nm, and the sheet resistance is considered approximately 10 ohm/sq based on the comparison with the commercial SnO_2_:F substrate. The transmittance rates of these samples are plotted in Fig. 6. The HF 10% and HF 25% etched glass samples show similar transmittance rates as the SnO_2_:F substrate (*T*_*400∼800 nm*_: 80.34%), although the transmittance of the HF 35% etched glass (*T*_*400∼800 nm*_: 77.16%) is lower than those of the other samples. The haze ratio of the HF 35% sample is higher than those of the other samples owing to a low specular transmittance, as shown in Fig. 6(c). The total transmitted light consists of the directly transmitted and diffused components. The specimen (HF 35% etched glass) exhibits high haze, which is influenced by wide-angle scattering. Diminished specular transmittance can be caused by reflecting a very rough and steep surface with an irregular height. This indicates that the light cannot be effectively transmitted owing to structures with steeper and roughly surface textures, which disappear and lower specular transmittance [19].

**Fig. 7:**
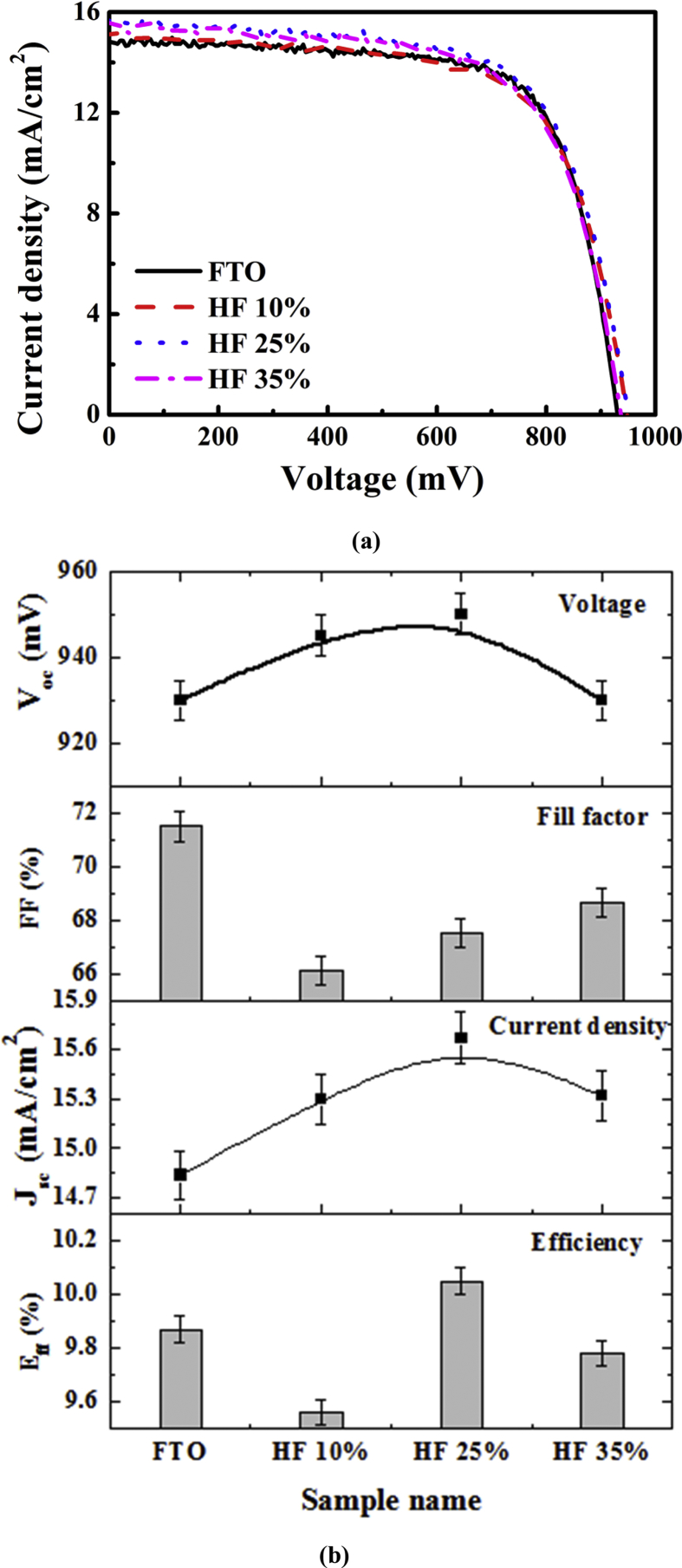
(a) Current-Voltage curves, and (b) solar cell parameters of the thin-film Si solar cells fabricated using a texture-etched glass substrate.

The solar cells fabricated using the texture-etched glass substrates are subjected to illuminated I–V studies, and the obtained solar cell parameters are presented in Fig. 7. The cell parameters, i.e., V_oc_, FF, and J_sc_, are improved by 20 mV, 0.14%, and 0.83 mA/cm^2^, respectively, for the SnO_2_:F substrate compared with the HF 25% samples. It is evident that the J_sc_ and FF were improved and resulted in the improvement of the overall efficiency of the analogous periodic-texture-etched glass. For an increased FF upon rougher glass surface with HF 35% etching, which is related to the increase in the parallel resistance and the decrease in the series resistance [20, 21], as shown in Table 1. During the deposition, the thickness of the amorphous silicon layers depends on the multi-scale surface (TCO film/textured glass) [22]. Therefore, it has an impact on the electrical properties with regard to the multi-scale surface.

**Table 1:** Diode parameters including the reverse saturation current density (J_0_), parallel resistance (R_P_), and series resistance (R_S_), the ideal diode factor (n) extracted from dark I-V curve characteristics.

Sample	J_0_ (A/cm^2^)	*R*_P_ (Ω cm^2^)	*R*_S_ (Ω cm^2^)	n
FTO	3.01 × 10^−8^	4141	1.2 × 10^−13^	3
HF 10%	1.93 × 10^−9^	1618	8	2.5
HF 25%	7.23 × 10^−10^	1933	9.4	2.34
HF 35%	2.08 × 10^−8^	3108	2.7 × 10^−13^	3.0

**Fig. 8:**
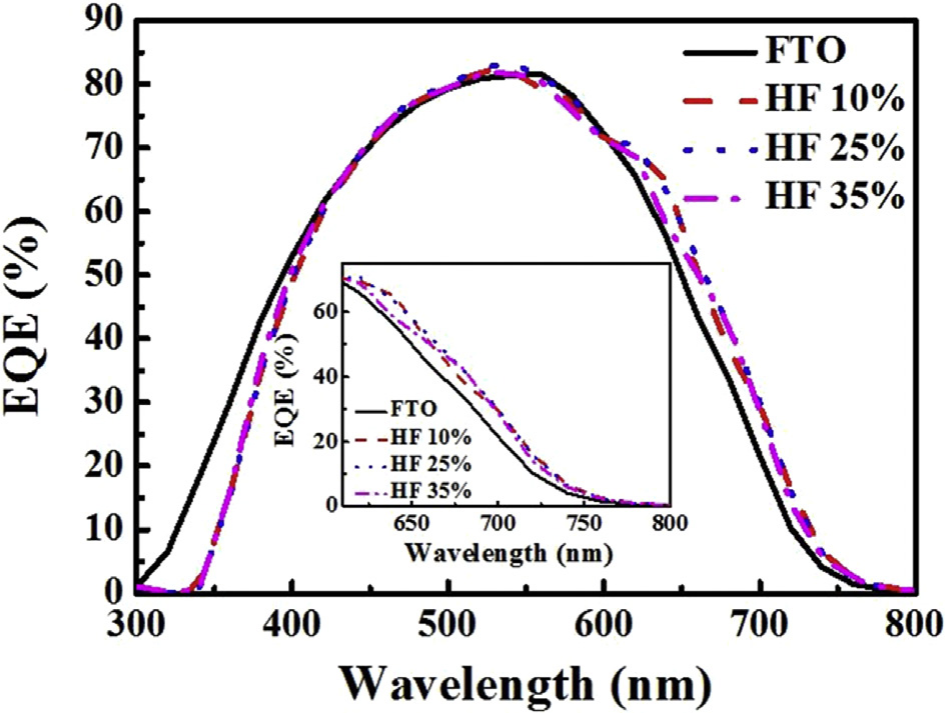
External quantum efficiency of the thin-film solar cell fabricated using a texture-etched glass substrate compared with that of the cell fabricated with the SnO_2_:F substrate. The inset graph is an enlarged view for the wavelength range 600 nm to 800 nm corresponding to the longer-wavelength region.

Fig. 8 shows the EQE of the TF solar cell fabricated using the texture-etched glass substrate compared with that of the cell fabricated with the SnO_2_:F substrate. To evaluate the J_sc_ gain of the cells, the current density is calculated from the spectrum irradiance at a given wavelength from 540 nm to 800 nm, and the results are as follows: HF 10%–9.38 mA/cm^2^, HF 25%–9.5 mA/cm^2^, HF 35%–9.3 mA/cm^2^, and FTO – 8.84 mA/cm^2^. All the TF solar cells deposited on the analogous periodic-texture-etched glass show a higher red response compared with the cells on the SnO_2_:F substrate; this increase is mainly due to the greater amount of light scattered by these substrates in the red part of the solar spectrum, as shown in the inset graph of Fig. 8. The maximum J_sc_ increase is observed for the HF 25% cell compared with other cells; this additional increase is attributed to the proper front-textured surface, which originates from the formation of shallow craters after the glass-etching process. This morphology, therefore, induces more scattering at the front side of the cell and leads to an improvement of the current-density gain for the HF 25% cell.

## 4 Conclusion

A geometrical hemisphere-array structure was developed with a textured-glass substrate using the HF-H_2_SO_4_ wet-etching method. The AZO films deposited on the textured glass were used for the fabrication of a-Si:H single-junction solar cells. The AZO thickness was 560 nm for optimal light trapping to avoid front and interference reflections. The transmittance rates of the sputtered-AZO films exhibited were similar to those of the FTO substrates in the cases of HF 10% and HF 25% samples. The hemisphere-array textured glass was improved by replacing the FTO substrates, and more effective light trapping was consequently achieved in the longer-wavelength region. By using the proposed method, improvements of the V_oc_ and J_sc_, which were, respectively, 20 mV and 0.83 mA/cm^2^ higher than those of the FTO substrates, were achieved.

## Declarations

### Author contribution statement

Hyeongsik Park: Conceived and designed the experiments; Analyzed and interpreted the data; Wrote the paper.

Doyoung Kim, Anh Huy Tuan Le, Junsin Yi: Analyzed and interpreted the data; Wrote the paper.

Junhee Jung, Duy Phong Pham, Jaehyun Cho: Performed the experiments.

Shahzada Qamar Hussain: Contributed reagents, materials, analysis tools or data.

### Funding statement

This work was supported by the New & Renewable Energy Core Technology Program of the Korea Institute of Energy Technology Evaluation and Planning (KETEP), granted financial resource from the Ministry of Trade, Industry & Energy, Republic of Korea. (No. 2 0163010012230).

### Competing interest statement

The authors declare no conflict of interest.

### Additional information

No additional information is available for this paper.
